# Durability of Functional SARS-CoV-2-Specific Immunological Memory and T Cell Response up to 8–9 Months Postrecovery From COVID-19

**DOI:** 10.1155/jimr/9743866

**Published:** 2025-02-10

**Authors:** Diptee Trimbake, Dharmendra Singh, Yogesh Gurav K., Prasad Babar, Varsha Dange S., Anuradha S. Tripathy

**Affiliations:** ^1^Department of Dengue and Chikungunya, Indian Council of Medical Research-National Institute of Virology, 20-A, Dr Ambedkar Road, Pune 411001, India; ^2^Department of Medicine, Pimpri Chinchwad Municipal Corporation, Pimpri, Pune 411018, Maharashtra, India

**Keywords:** antibody, COVID-19, ELISPOT, flow cytometry, memory B and T cells, recovered individuals, T cell response

## Abstract

Research on long-term follow-up in individuals who have recovered from coronavirus disease-19 (COVID-19) would yield insights regarding their immunity status and identify those who need booster vaccinations. This study evaluated the longevity of severe acute respiratory syndrome coronavirus 2 (SARS-CoV-2)-specific cellular and humoral memory responses, as well as T cell effector functionalities, at 1–2 months (*n* = 40), 8–9 months (*n* = 40), and 12 months/1 year (*n* = 27) following recovery from SARS-CoV-2 infection. CTL response by enzyme-linked immunospot (ELISPOT); levels of cytokine by Bio-Plex, natural killer (NK), CD4+ helper, and CD8+ cytotoxic T cell functionalities using flow cytometry; anti-SARS-CoV-2 IgG by ELISA; and levels of neutralizing antibodies (NAbs) by surrogate virus NAb assay were assessed. The levels of SARS-CoV-2-specific IgG and NAb at 1–2 and 8−9 months postrecovery were hand in hand and appeared declining. SARS-CoV-2-specific B, memory B and plasma cells, and T cells sustained up to 8–9 months. Increased expression of CD107a/IFN-γ by NK cells and cytotoxic T cells at 8–9 months could be indicative of SARS-CoV-2-specific effector functions. Recovered individuals with positive and negative IgG antibody status displayed T cell response up to 1 year and 8–9 months, respectively, emphasizing the durabilty of effector immunity up to 8–9 months regardless of IgG antibody status. Overall, the recovered individuals exhibited robust immunological memory, sustained T cell response with effector functionality against SARS-CoV-2 that persists for at least 8–9 months.

## 1. Introduction

Severe acute respiratory syndrome coronavirus 2 (SARS-CoV-2) sparked the global coronavirus disease-19 (COVID-19) pandemic. Various effective vaccine platforms are developed for controlling the pandemic [1]. However, the introduction of new variants of interests (VoIs) and variants of concerns (VoCs) may present a heightened risk to public health globally. Consequently, it is essential to investigate and comprehend the longevity of the protective immunological memory response to SARS-CoV-2 following natural infection or recovery [2]. The existance of antispike or antinucleocapsid IgG antibodies was associated with a substantially reduced risk of SARS-CoV-2 reinfection within 6 months among seropositive and seronegative healthcare professionals [3, 4]. A study in the Indian population detected receptor binding domain (RBD) and N protein-specific IgG antibodies up to 240 days postonset days of illness, which correlated with virus neutralization response. Detection of neutralizing antibodies (NAbs) in COVID-19 patients with high variation in titers and without real correlation with clinical courses is also reported [5]. Studies carried out in acute and convalescent COVID-19 patients have indicated that the presence of T cell responses are associated with mild disease, implying that SARS-CoV-2-specific CD4+ helper T and CD8+ cytotoxic T cell responses may be crucial for control and resolution of primary SARS-CoV-2 infection [6–10].

SARS-CoV-2 vaccination aimed to build effective population-level immunity to mitigate the transmission of COVID-19. In this context, data regarding the efficacy and longevity of protective immunity postvaccination and during primary infection with SARS-CoV-2 are critical [11]. Dan et al. [12] have reported that about 93% of participants at 1 month and 92% at 6–8 months post-COVID-19 had CD4+ T cell memory response. An earlier study carried out in patients with ongoing COVID-19 infection and in recovered individuals suggested that the immunological reaction triggered by SARS-CoV-2 is mainly T lymphocyte-mediated [12]. Previous research revealed higher CD4+ helper T/Th memory, CD8+ cytotoxic T/Tc memory, and B memory cells in the recovered individuals at 45–60 days postrecovery compared to mild symptomatic patients and suggested that it could be proposed as markers/indicators of recovery from mild infection [13]. Emerging research indicates a robust memory B cell response in people rescued from COVID-19 [11, 12]. Memory T and B cells with the functional ability to generate antibodies immediately upon reintroduction of a pathogen may act as surrogates of protection in turn lending continuous protective immunity [14, 15]. Currently, the consensus is that antibodies are insufficient predictors of protection, whether following immunization or infection. Consequently, extended follow-up studies featuring a comprehensive analysis of the durability of SARS-CoV-2-specific B and T cell responses and serum IgG antibodies as well as NAb responses in naturally recovered individuals from SARS-CoV-2 infection would yield insights into the protective effect of recovery from prior infection.

The current study has evaluated the durability of SARS-CoV-2-specific B and T cell responses, serum IgG antibodies, and surrogate NAb responses in naturally recovered individuals at 1–2 months (*n* = 40), 8 months (*n* = 40), and 1 year (*n* = 27) postrecovery from SARS-CoV-2 infection from Pune, Maharashtra, India.

## 2. Materials and Methods

### 2.1. Study Subjects

The current follow-up study was carried out between June 2020 and June 2021. The recovered individuals had a previous history of mild symptomatic/asymptomatic SARS-CoV-2 infection and were mostly distinct. The blood samples were collected at 1–2 months (*n* = 40), 8–9 months (*n* = 40), and 1 year (*n* = 27) postrecovery.

For ease of reading, recovered individuals at 1–2 months, 8–9 months, and 1 year postrecovery are termed as R1, R2, and R3, respectively. This terminology is followed in the material method and result sections.

It is important to note that individuals at R1, R2, and R3 were negative for SARS-CoV-2 RT-PCR. This is to note that five individuals at R3 were reported to be vaccinated with first dose of SARS-CoV-2, hence removed from further analysis. A limited number of participants were lost due to logistical obstacles such as work and family commitments, geographic mobility, transportation issues, and other factors.

Fifty-six healthy particiapants were recruited from the blood donation camps hosted by Sassoon General Hospital, Pune, in December 2020. These healthy controls tested negative for anti-SARS-CoV-2 IgG antibody (COVID Kavach, ELISA, M/s Cadila Healthcare Limited, Ahmedabad).

The Institutional Ethical Committee for Research on Humans at National Institute of Virology, Pune, approved the study in accordance with the standards established by the Indian Council of Medical Research, New Delhi (NIV/IEC/May/2020/D-5, May 26, 2020). Informed consents were provided by all the study participants, and the participants had agreed upon for use of their data in the research. All samples were processed according to Institutional Biosafety Guidelines. The study design is illustrated in Figure S1.

### 2.2. Isolation of Peripheral Blood Mononuclear Cells (PBMCs), Plasma Separation, and Storage

Freshly collected 3–4 mL of peripheral blood from the study participants was processed for isolation of PBMCs by density gradient centrifugation technique [16–20]. Briefly, the whole blood was carefully layered onto Ficoll-Hypaque (Sigma, USA) in a 15-mL centrifuge tube (Tarsons, India) and spun at 2000 rpm for 30 min at room temperature (RT). The plasma layer on the top was removed slowly, aliquoted, and stored at −80°C until use. The opaque layer was aspirated which was present below the plasma layer and put into a tube containing Roswell Park Memorial Institute (RPMI) 1640 medium (Gibco, USA), followed by centrifugation at 1000 rpm for 10 min. If red blood cells (RBCs) were present, the pellet was lysed with 1× RBC lysis buffer for 10 min at RT. Two successive washes were given with RPMI 1640 medium, as described in the previous step. A viable cell count was done using the trypan blue dye exclusion method. A final cell suspension of 1 × 10^6^ cells/mL was made using complete RPMI culture medium (RPMI + 10% fetal bovine serum [FBS]) (Gibco, USA) [13, 16–20]. The plasma samples were preserved at −80°C and thawed at the time of identification of anti-SARS-CoV-2 IgG antibody by ELISA and assessment of NAbs against SARS-CoV-2 using surrogate virus neutralization (sVNT) assay (cPass, GenScript USA).

### 2.3. Flow Cytometry Analysis

**Figure 1:**
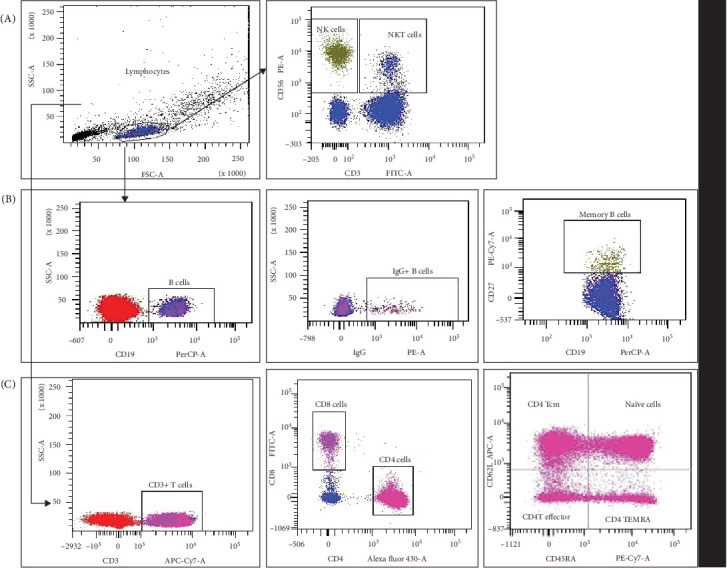
Gating strategy to distinguish different lymphocyte populations by flow cytometry. The peripheral blood mononuclear cells (PBMCs) from the study participants were stained with panels of fluorochrome-labeled antibodies to assess the frequency and immune profile. The numbers in the histogram are the mean of the cell population representing for the study group. (A) Lymphocytes and natural killer (NK cells [CD3–CD56+]), natural killer-like T (NKT-like [CD3+CD56+]) cell profile. (B) B (CD19+), IgG+ B cells, and memory B (CD19+CD27+) cell profile. (C) Helper T (CD3+CD4+), cytotoxic T (CD3+CD8+) cell profile, memory Th and Tc cells, CD4+ naïve cells, CD4+ T central memory cell effector cells, and terminally differentiated effector memory cells (TEMRA) cells.

1 × 10^6^ freshly isolated PBMCs were utilized for surface staining of natural killer (NK), natural killer-like T (NKT), B, T, memory B, and T cells using cocktail of antibodies (anti-CD19 PerCP-Cy5.5 [clone HIB-19], anti-CD27 PECy7 [clone M-T271], anti-IgG PE [clone G18-145], anti-CD3 APCH7 [clone SK7], anti-CD4 BV480 [clone SK3], anti-CD8 FITC [clone RPA-T8], CD45RA PECy7 [clone HI 100], anti-CD62L APC [clone DREG-56], CCR7 PE [clone 2-L1-A], and NK Tritest [anti-CD3 FITC, clone SK7; anti-CD56 PE, clone NCAM 16.2; anti-CD16 PE, clone B73.1; and anti-CD45 RA, L48] [ASR], all from BD Biosciences, San Jose, CA) following a previously established protocol [21–23]. Briefly, PBMCs were incubated with antihuman monoclonal antibodies for 30 min at room temperatures in the dark conditions. Subsequent to washing, the cells were fixed with 2% paraformaldehyde, acquired on FACS Aria II (BD Biosciences). Each experiment involved acquisition of 50,000 events within the lymphocyte gate accompanied by the relevant isotype control. FACS Diva software (Becton Dickinson, USA) was used for data analysis, and results are presented as the percentage of positive cells in the gated population [13, 16–20]. The strategy for gating is provided in Figure 1.

#### 2.3.1. SARS-CoV-2 S1-Stimulated B, T, Memory B, and T Cell Enumeration

1 × 10^6^ freshly isolated PBMCs were cultured with/without SARS-CoV-2 S1 protein/antigen (10 µg/mL, SARS-CoV-2 [2019-nCoV] spike S1 [D614G]-His Recombinant Protein, Sino Biological, China) in RPMI1640 medium with 10% FBS for 48 h. The cells were harvested postincubation and surface stained with cocktail of antibodies (anti-CD19 PerCP-Cy5.5 [clone HB-19], anti-CD27 PECy7 [clone M-T271], anti-IgG PE [clone G18-145], anti-CD3 APCH7 [clone SK7], anti-CD4 BV480 [clone SK3], anti-CD8 FITC [clone RPA-T8], CD45RA PECy7 [clone HI 100], anti-CD62L APC [clone DREG-56], and CCR7 PE [clone 2-LI-A]) for 30 min at 4°C in the dark coditions. The cells were fixed with 2% paraformaldehyde and acquired on FACS Aria II (BD Biosciences) postwashing procedure [16–20].

### 2.4. Intracellular Cytokine Staining

**Figure 2:**
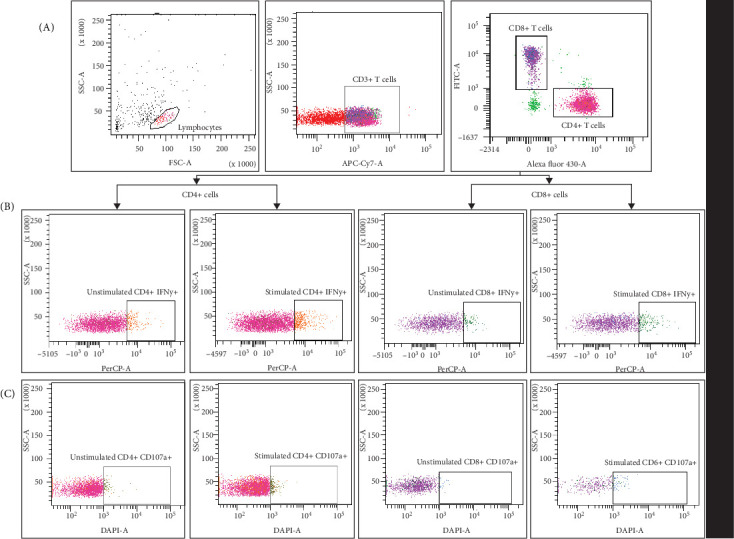
Gating strategy to distinguish different lymphocyte populations and intracellular cytokine expression by flowcytometry. IFN-γ and CD107a produced by CD4+ T cells and CD8+ T cells. T cells were derived from the isolated peripheral blood mononuclear cells (PBMCs) gated by forward and side scatter. The numbers in the histogram are the mean of the cell population representing the study group. (A) Lymphocytes and T helper (CD3+CD4+), T cytotoxic (CD3+CD8+) cell profile. (B) Percentage of IFN-γ produced by CD4+ T and CD8+T cells according to the fluorescence of each cytokine in unstimulated and stimulated cells. (C) Percentage of CD107a produced by CD4+ T and CD8+ T cells according to the fluorescence of each cytokine in unstimulated and stimulated cells.

The functional capacity of NK, CD4+ helper T cells, and CD8+cytotoxic T cells were assessed by intracellular expression of IFN-γ/CD107a using multicolor flow cytometry as before [23]. Briefly, freshly isolated PBMCs (1.0 × 10^6^) were stimulated with SARS-CoV-2 S1 protein (10 µg/mL, Sino Biological) in the presence of anti-CD107a BV421 (clone H4A3, BD Biosciences) for 1 h at 37°C in 5% CO_2_, followed by incubation with Brefeldin A (10 µg/mL; Sigma Aldrich, USA) and GolgiStop (monensin, 1.5 µg/mL; BD Biosciences) for 5 h. Unstimulated cells were also included to evaluate the background/baseline response. The cells were harvested after stimulation and incubated with a cocktail of surface antibodies (anti-CD3 APCH7 [clone SK7], anti-CD4 BV480 [clone SK3], anti-CD8 FITC [clone RPA-T8], anti-CD56 APC [clone B159 (RUO)], BD Biosciences) for 30 min at 4°C in the dark conditions. The cells were fixed and permeabilized with cytofix/cytoperm buffer (BD Biosciences) according to manufacturer's instruction and processed for intracellular staining with IFN-γ BB700 antibodies ([clone B27] [BD Biosciences]) for 30 min in the dark enviromnment. The cells were washed and stored at 4°C in the dark until acquisition on FACS Aria-II and analyzed using FACS Diva software version 8 (BD Biosciences). Single-color compensation was conducted prior to acquisition of samples in the FACS Aria II flow cytometer. For each experiment, 50,000 events were acquired within the lymphocyte gate with appropriate isotype control. Data were analyzed using FACS Diva software (BD Biosciences) [16–20]. The gating strategy is depicted in Figure 2.

### 2.5. SARS-CoV-2-Specific IFN-γ Release by Enzyme-Linked Immunospot (ELISPOT)

To quantify the number of SARS-CoV-2 S1-specific IFN-γ-secreting spot-forming cells (SFCs), 1 × 10^5^ viable PBMCs were stimulated with SARS-CoV-2 S1 protein (10 µg/mL, Sino Biological) for 48 h. Cells devoid of any antigen functioned as negative controls, while cells treated with 10 µg/mL of phytohemagglutinin (Sigma Aldrich, USA) acted as positive controls. All assays were carried out in triplicates. The IFN-γ SFCs were quantified on an ELISPOT reader, customized software (AID GmbH, Strassberg, Germany) and were represented as the number per 10^5^ cells [2, 16–20].

### 2.6. Stimulation of PBMCs and Cytokine Assay

5 × 10^5^ PBMCs were stimulated with SARS-CoV-2 S1 antigen (10 µg/mL, Sino Biological) and were cultured at 37°C, 5% CO_2_ for cytokine assay. Supernatants from cultured cells were harvested at 72 h and stored at −80°C. Cytokine concentrations in the SARS-CoV-2 S1-stimulated culture supernatants were measured on the Bio-plex Multiplex Immunoassay System (Bio-Rad, Hercules, CA, USA) using a Bio-plex Pro Human Cytokine 27-plex assay kit utilized according to the manufacturer's guidelines as previously documented [24]. The levels of 15 cytokines including the proinflammatory (IL-1β, IL-5, IL-6, IL-7, IL-9, IL-15, IL-17, and TNF-α), anti-inflammatory (IL1-RA, IL-4, IL-10, and IL-13), and Th1 cytokines (IL-2, IFN-γ, and IL-12p70) along with 7 chemokines (eotaxin, CCL-2, CCL-3, CCL-4, CCL-5, IL-8, and CXCL-10) and 5 growth factors (basic fibroblast growth factor [FGF], G-CSF, GM-CSF, vascular endothelial growth factor [VEGF], and platelet-derived growth factor-bb (PDGF-bb]) were estimated. The lowest value of the respective standards was utilized for undetectable quantities/concentrations of the cytokines, chemokines, and growth factors in the analyzed samples [21].

### 2.7. Software and Statistical Analyses

The statistical analyses were conducted on IBM SPSS Statistics 25 software (SPSS Inc., Chicago, IL, USA) and GraphPad Prism 8 software (GraphPad, San Diego, CA, USA). The Mann–Whitney *U* test was employed for the comparison among the recovered groups. The mean of triplicate experiments in ELISPOT assay was utilized for the analysis. Changes in parameter over the time (SARS-CoV-2 recovery duration) were analyzed with Wilcoxon signed-rank sum test. All the data are presented as median (IQR). A *p* value less than 0.05 was deemed significant. Concentrations all analytes/cytokines were assessed using measured levels of specific cytokines. The Bonferroni's correction was implemented to account multiple comparisons for each analyte/cytokine. Thus, the traditional cutoff for *p* value (0.05) was adjusted/lowered down by dividing it by the number of comparisons made for each analyte and considered *p* value as 0.008 for cytokines comparison. The dot plots were created using GraphPad Prism 8 software (GraphPad, San Diego, CA, USA). The dots signify the individual values, and the bars indicate mean + SD values.

## 3. Results

### 3.1. Characteristics of the Study Population

Table 1 illustrates the characteristics of the study groups. The recovered individuals had previous history of SARS-CoV-2 infection and were negative for SARS-CoV-2 RT-PCR at the time of enrollment and included (a) 40 recovered individuals at 1–2 months. Among them, 24 (60%) and 16 (40%) had recovered from asymptomatic and symptomatic infections, respectively. Notably, 26 (65%) were anti-SARS-CoV-2 IgG+ as tested by ELISA (b) 40 recovered individuals at 8–9 months postrecovery. Among them, 22 (55%) and 18 (45%) had recovered from asymptomatic and symptomatic infections, respectively. Twenty-one (52.5%) were anti-SARS-CoV-2 IgG+ (c) 27 recovered individuals at 1 year postrecovery. Recovered individuals at 1−2 and 8−9 months postrecovery did not report any history of reinfection and/or vaccination, whereas 5 of 27 recovered individuals at 1 year postrecovery had reported first dose of vaccination. Hence, 22 recovered individuals 1 year postrecovery were only considered for the analysis. Among them, 12 (54.5%) and 10 (45.4%) had recovered from asymptomatic and symptomatic SARS-CoV-2 infections, respectively. Notably, 17 out of 22 (77.2%) were anti-SARS-CoV-2 IgG+ by ELISA, and (d) 56 healthy controls who were anti-SARS-CoV-2 IgG− did not report any history of SARS-CoV-2 infection/and vaccination.

### 3.2. Flow Cytometry Analysis

#### 3.2.1. Percentages of NK and NKT Cells in Recovered Individuals

**Figure 3:**
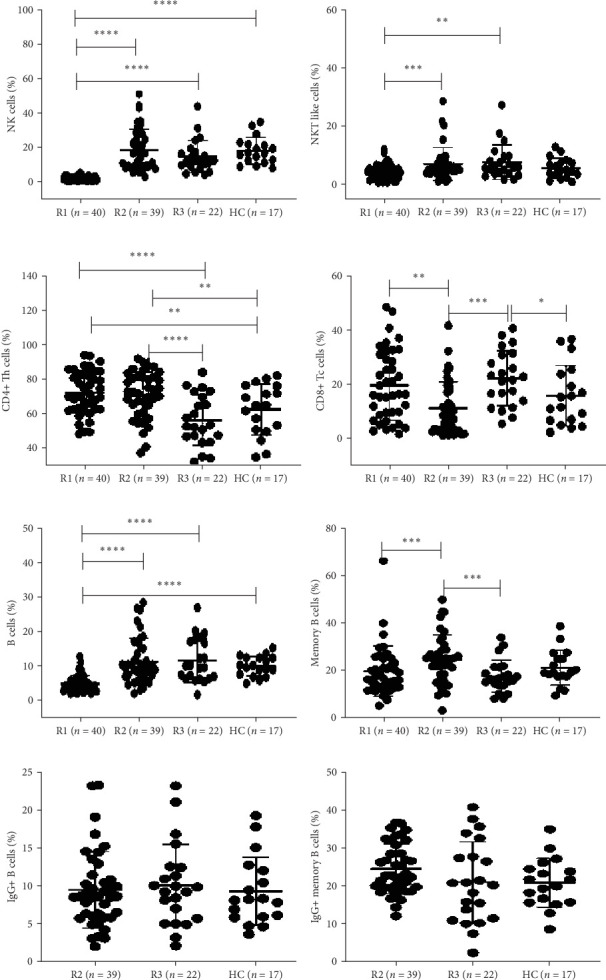
Peripheral blood mononuclear cells (PBMCs) from recovered individuals R1: post 1–2 months (*n* = 40), R2: 8–9 months (*n* = 40) and R3:12 months/1 year post recovery (*n* = 22) and HC: healthy controls (*n* = 17) were stained and acquired on flowcytometer. Vertical scatter plots denote the comparisons of frequencies of immune cells and their subpopulation among different study groups: (A) natural killer (NK) cells (B) natural killer T (NKT) cells (C) CD4+ T helper cells (D) CD8+ T cytotoxic cells profile (E) B cells (F) Memory B cells (G) IgG+ B cells post 8–9 months and 12 months/1 year (H) IgG+ memory B cells post 8–9 months and 12 months/1 year. Data are presented as the percentage of immune cells out of lymphocytes. The dots represent individual values and bars represent mean + SD values. (*⁣*^*∗*^*p*-value <0.05, *⁣*^*∗∗*^*p*-value <0.005, and *⁣*^*∗∗∗*^*p*-value <0.0001).

The percentages of NK cells were significantly lower in SARS-CoV-2-recovered individuals at R1 compared to healthy controls (NK cells R1, 2.2 [1.3–3.27]; HCs, 16.7 [12.2–22.05] *p* < 0.0001). The percentages of both NK and NKT cells were significantly lower in SARS-CoV-2-recovered individuals at R1 compared to recovered groups at R2 and R3 (NK cells R1, 2.2 [1.3–3.27]; R2, 16.5 [9.25–26.9]; R3, 12.4 [8.9–17.8]; NKT cells R1, 4.2 [2.2–5.85]; R2, 5.8 [4.0–7.47]; R3, 5.8 [3.75–9.55], respectively, *p* < 0.05 in each) (Table 2, Figure 3A,B).

#### 3.2.2. Percentages of B and Memory B Cells in SARS-CoV-2-Recovered Individuals

The percentages of B cells were significantly higher in SARS-CoV-2-recovered individuals at R2 and R3 compared to recovered individuals at R1 (B cells R1, 4.5 [3.4–5.8]; R2, 9.4 [6.2–14.5]; R3, 9.85 [6.5–16.9]; HCs, 9.9 [7.5–12.4], respectively, *p* < 0.05 in each) (Table 2, Figure 3E). The percentages of memory B cells were significantly higher at R2 compared to R1 and R3 (memory B cells R1, 17.2 [12.8–24.5]; R2, 24.7 [16.5–30.3]; R3, 16.7 [13.9–21.43], respectively, *p* < 0.05 in each) (Table 2, Figure 3F). However, the percentages of IgG+ B cells and IgG+ memory B cells were comparable at the time points for which data were available (Table 2, Figure 3G,H).

#### 3.2.3. Percentages of T and Memory T Cell Subsets in SARS-CoV-2-Recovered Individuals

**Figure 4:**
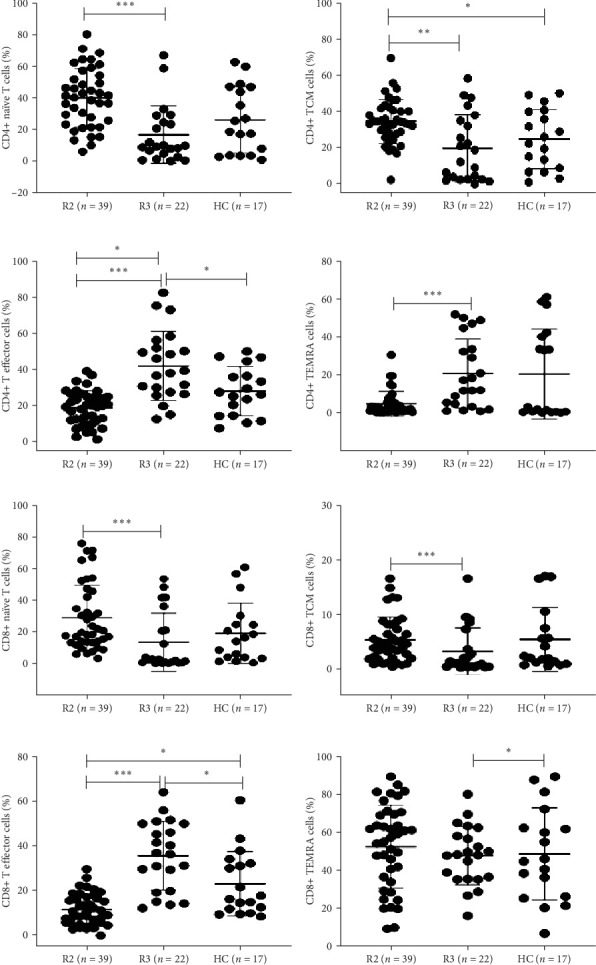
Flow cytometric analysis of T and memory T cell subsets in SARS-CoV-2-recovered individuals. Peripheral blood mononuclear cells (PBMCs) from recovered individuals R2, 8–9 months (*n* = 40); R3, 12 months/1 year postrecovery (*n* = 22); and healthy controls (HCs) (*n* = 17) were stained and acquired on flowcytometer. Vertical scatter plots denote the comparisons of frequencies of immune cells and their subpopulation among different study groups: (A–D) CD4+ memory T cell subsets and (E–H) CD8+ memory T cell subsets, namely, naive, central, terminally differentiated effector memory cells (TEMRA), and effector memory cells post 8–9 months and 12 months/1 year. Data are presented as percentage of immune cells out of lymphocytes. The dots represent individual values, and the bars represent mean + SD values (*⁣*^*∗*^*p* value <0.05, *⁣*^*∗∗*^*p* value <0.005, and *⁣*^*∗∗∗*^*p* value <0.0001).

The percentage of CD4+ T cells were significantly higher at R1 and R2 compared to R3 and healthy controls (CD4+ T cells R1, 71.4 [63.3–83.2]; R2, 73.3 [62.78–81.65]; R3, 53.7 [46.63–71.18]; HCs, 65.6 [51.2–76.85], respectively, *p* < 0.05 in each) (Table 2, Figure 3C). The percentages of CD8+ T cells were significantly higher at R1 and R3 compared to R2 (CD8+ T cells R1, 16.4 [9.1–30.53]; R2, 8.7 [3.02–17.48]; R3, 22.2 [13.38–30.95], respectively, *p* < 0.05) (Table 2, Figure 3D). At the memory compartment, the percentages of CD4+ memory T cells, namely, naïve and central (*T*_CM_) memory T cells, were significantly higher at R2 compared to R3 and healthy controls (CD4+ naïve T cells R2, 41.1 [24.23–53.9]; R3, 9.7 [4.65–26]; HCs, 22.4 [3.95–47.3]; CD4+ [*T*_CM_] R2, 34. 2 [28–41.53]; R3, 15.7 [3.17–35.85]; HCs, 24.4 [8.65–40.1, *p* < 0.05 in each]) (Table 2, Figure 4A,B). CD4+ terminally differentiated effector memory cells (TEMRAs) and CD8+ TEMRAs were significantly lower at R2 compared to R3 and healthy controls, respectively (CD4+ TEMRA R2, 19.4 [11.98–24.9]; R3, 38.4 [27.03–52.68]; HCs, 26.6 [14.35–38.25]; CD8+ TEMRA R2, 11 [5.97–17.43]; R3, 36.8 [19.95–50]; HCs, 17.3 [11.83–32.88], *p* < 0.05 in each) (Table 2, Figure 4D,H). CD8+ memory T cells, namely, naïve T cells and central (*T*_CM_), were significantly higher at R2 compared to R3 (CD8+ naïve T cells R2, 20 [13.3–44.5]; R3, 2.4 [0.55–24.65]; CD8+ (*T*_CM_) R2, 3.95 [1.7–7.67]; R3, 0.8 [0.2–4.3], *p* < 0.05 in each) (Table 2, Figure 4E,F).

CD4+ and CD8+ effector T cells were significantly lower at R2 compared to R3 and healthy controls (CD4+ effector T cells R2, 2.3 [1.3–6.07]; R3, 14.9 [4.625–36.48]; HC, 2.7 [0.7–40.8]; CD8+ effector T cells R2, 56.35 [34.95–69.35]; R3, 48.4 [35.85–59.28]; HC, 45.6 [26.5–64.8], *p*  < 0.05 in each).

#### 3.2.4. Percentages of B and Memory B Cells in SARS-CoV-2-Recovered Individuals Poststimulation With SARS-CoV-2 S1 Protein

**Figure 5:**
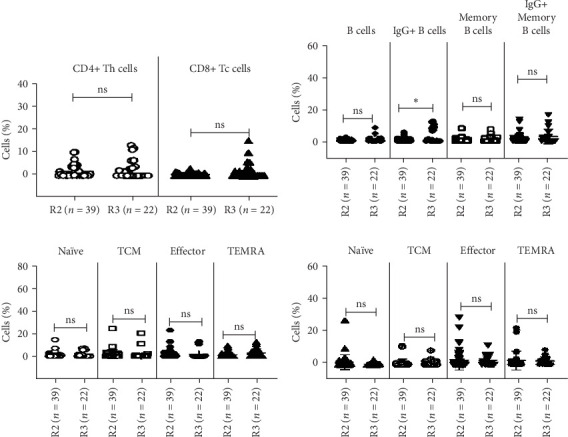
Flowcytometric analysis of SARS-CoV-2 S1-stimulated memory T, B, and IgG+ B cells in SARS-CoV-2-recovered individuals. Peripheral blood mononuclear cells (PBMCs) from the recovered individuals R2, 8–9 months (*n* = 40), and R3, 12 months/1 year postrecovery (*n* = 22), were stimulated with SARS-CoV-2 S1antigen for 48 h and then stained and acquired on flowcytometer. Vertical scatter plots denote the comparisons of frequencies of immune cells and their subpopulation among different study groups: (A) CD4+ helper T/Th and CD8+ cytotoxic/Tc memory T cell profile, (B) B cells and memory B cell subsets, (C) CD4+ helper memory T cell profile, and (D) CD8+ cytotoxic memory T cell profile post 8–9 months and 12 months/1 year. Data are presented as percentage of immune cells out of lymphocytes. The dots represent individual values, and the bars represent mean + SD values (*⁣*^*∗*^*p* value <0.05, *⁣*^*∗∗*^*p* value <0.005, and *⁣*^*∗∗∗*^*p* value <0.0001). ns, nonsignificant.

The percentages of SARS-CoV-2 S1-simulated B and memory B cells were significantly higher at R2 compared to healthy controls (SARS-CoV-2 S1-simulated B cells R2, 0.1 [0–0.9]; HCs, 0 [0–0.10], *p* < 0.05; SARS-CoV-2 S1-simulated memory B cells R2, 0.2 [0–1.2]; HCs, 0 [0–0], respectively, *p* < 0.05 in each) (Table 3). The percentages of SARS-CoV-2 S1-simulated IgG+ B cells were significantly higher at R2 and R3 compared to healthy individuals (SARS-CoV-2 S1-simulated IgG+ B cells R2, 0.2 [0–1.2]; R3, 1 [0–9.0]; HCs, 0 [0–0.07], *p* < 0.05 in each) (Table 3 and Figure 5B).

Among the groups, the percentages of SARS-CoV-2 S1-simulated memory B cells and IgG+ memory B cells were comparable at R2 and R3 (Table 3 and Figure 5B).

#### 3.2.5. Percentages of Memory T Cells and Subsets, in SARS-CoV-2-Recovered Individuals Poststimulation With SARS-CoV-2 S1 Protein

Among the groups, the percentages of SARS-CoV-2 S1-simulated CD4+ Th, CD8+ Tc, and memory T subsets, namely, naïve T cells, central (*T*_CM_), and effector (*T*_EM_) memory cells and TEMRAs, were comparable at R2 and R3 (Table 3 and Figure 5A,C,D).

These observations indicate that B and memory B cells persist up to 8–9 months postinfection/recovery, in agreement with the status of T cell and humoral response.

#### 3.2.6. Persistence of T and NK Cell Effector Response Post-SARS-CoV-2 Infection

The ability of SARS-CoV-2 S1-stimulated CD4+, CD8+ T, and NK cells from the COVID-19-recovered groups and healthy individuals to express IFN-γ/CD107a as a marker of cytotoxicity was assessed in flowcytometry. The percentages of IFN-γ/CD107a-expressing CD8+ T cells were significantly higher at R2 compared to R3 and healthy controls (CD8+ IFN-γ+ cells R2, 0.9 [0.50–2.3]; R3, 0 [0.00–0.65]; HCs, 0.4 [0.1–1.1]; CD8+ CD107a+ cells R2, 2.7 [1.4–4.5]; R3, 0.8 [0.05–1.4]; HCs, 1.3 [0.6–1.8], respectively, *p* < 0.05 in each) (Table 4, Figure 5B).

**Figure 6:**
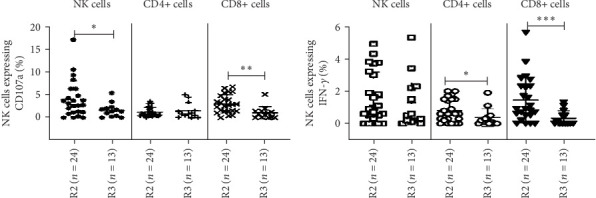
Flowcytometric analysis intracellular expression of SARS-CoV-2 S1-stimulated IFN-γ and CD107a in the recovered individuals. Peripheral blood mononuclear cells (PBMCs) from the recovered individuals R2, 8–9 months (*n* = 24), and R3, 12 months/1 year postrecovery (*n* = 13), were stimulated with SARS-CoV-2 S1 antigen for 6 h and then stained and acquired on flowcytometer. Vertical scatter plots denote the comparisons of frequencies of immune cells and their subpopulation among different study groups: (A) CD4+ T helper cells, CD8+ T cytotoxic cells, and natural killer (NK) cell profile with CD107a expression. (B) CD4+ T helper cells, CD8+ T cytotoxic cells, and NK cell profile with IFN-γ expression post 8–9 months and 12 months/1 year. Data are presented as the percentage of immune cells out of lymphocytes. The dots represent individual values, and the bars represent mean + SD values (*⁣*^*∗*^*p* value <0.05, *⁣*^*∗∗*^*p* value <0.005, and *⁣*^*∗∗∗*^*p* value <0.0001).

Similarly, the percentages CD107a-expressing NK cells were significantly higher at R2 compared to R3 and healthy controls (NK+ CD107a+ cells R2, 2.85 [1.13–5.83]; R3, 1.3 [0.30–2.15]; HCs, 1.1 [0.2–2.2], respectively, *p*  < 0.05) (Table 4, Figure 6A). The percentages of IFN-γ-expressing CD4+ T cells were higher at R2 compared to R3 (CD4+ IFN-γ cells R2, 0.6 [0.23–1.50]; R3, 0.2 [0.00–0.45]; HCs, 1.1 [0.2–2.2], respectively, *p*  < 0.05) (Table 4, Figure 6B). However, the percentages of IFN-γ-expressing NK cells and CD107a-expressing CD4+ cells were comparable at R2 and R3 (Table 4, Figure 6).

### 3.3. SARS-CoV-2 S1-Specific CTL Response in ELISPOT

We conducted an ELISPOT experiment utilizing recombinant S1 protein as a recall antigen to assess the SARS-CoV-2-specific IFN-γ response. In the control group (*n* = 16), IFN-γ responses in unstimulated, SARS-CoV-2 S1-stimulated, and PHA-stimulated cells were 0 (0–1.3), 1.6 (0–4.5), and 166 (102–192) SFCs/10^5^ cells, respectively. In the recovered group R1 (*n* = 34), IFN-γ responses in unstimulated, SARS-CoV-2 S1-stimulated, and PHA-stimulated cells were 2.85 (1–5.08), 9.6 (1.2–45.18), and 121 (84–191) SFCs/10^5^ cells, respectively. In the recovered group R2 (*n* = 39), IFN-*γ* responses in unstimulated, SARS-CoV-2 S1-stimulated, and PHA-stimulated cells were 0.7 (0.3–3.35), 2 (0.2–3.3), and 217 (101–541) SFCs/10^5^ cells, respectively. In the recovered group R3 (*n* = 22), IFN-γ responses in unstimulated, SARS-CoV-2 S1-stimulated, and PHA-stimulated cells were 0.8 (0.0–14.3), 2.6 (0–20.7), and 129 (1.3–500) SFCs/10^5^ cells, respectively. The number of SFCs in unstimulated wells was subtracted from the antigen-stimulated wells in each subject group for normalization of data. The cutoff level for SFCs was set as using the number of SFCs in the negative control wells. The test was scored as positive when (i) the test wells contained a mean SFCs count of at least 5 more SFCs than the mean of the negative control wells if the negative control wells reported 0–5 SFCs; (ii) test wells contained a mean of at least twice the mean number of SFCs of the negative control wells, if the negative control well reported 6–10 SFCs; and (iii) test wells contained a mean of at least three times the mean number of SFCs of the negative control wells if the negative control wells reported 11–20 SFCs. Positive control wells containing <20 SFCs/negative control wells containing >20 SFCs were considered as an invalid test result. Wells with a high background or without a PHA response were excluded.

#### 3.3.1. Magnitude and Strength of SARS-CoV-2 S1-Specific T Cell Response

**Figure 7:**
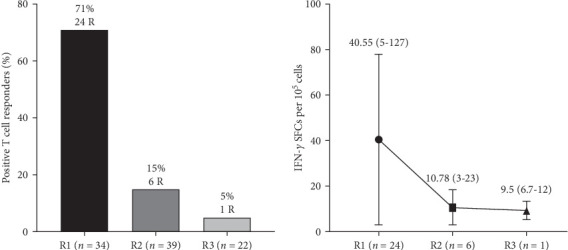
Strength and magnitude of SARS-CoV-2 S1-specific IFN-γ-producing T cell response in SARS-CoV-2-recovered individuals. Strength and magnitude of SARS-CoV-2 S1-specific IFN-γ-producing T cell response in different groups post 1–2 months (R1, *n* = 34), 8–9 months (R2, *n* = 39), and 1 year of recovery (R3, *n* = 22). Peripheral blood mononuclear cells (PBMCs) were isolated from all subjects mentioned and were cultured with SARS-CoV-2 S1 antigen protein in vitro. IFN-γ-secreting cell frequencies were determined by ELISPOT assay. (A) Magnitude and (B) strength of the SARS-CoV-2 S1-specific IFN-γ-producing T cell response in terms of percentage.

Overall, T cell response was observed in 24 out of 34 (71%) individuals at R1, 6 out of 39 (15.38%) individuals at R2, and in 1 out of 22 (5%) individuals at R3 (Figure 7B). A decrease in the magnitude of T cell response was observed over a period of time postrecovery (Figure 7A,B). The strength of T cell responses against S1 antigen (in terms of SFC/10^5^ cells) were 40.55 (5–127),10.78 (3–23), and 9.5 (6.7–12) at 1–2 months, 8–9 months, and 1 year of postrecovery, thus exhibiting an apparently decreasing trend with time postinfection (Figure 7B).

#### 3.3.2. SARS-CoV-2 S1-Specific T Cell Response in Anti-SARS-CoV-2 IgG+ Recovered Individuals

Thirteen out of 21 (62%), 5 out of 21(24%), and 1 out of 17 (6%) of anti-SARS-CoV-2 IgG+ recovered individuals demonstrated T cell response at post 1–2 months, 8–9 months, and 1 year postrecovery, respectively, indicating that though in an apparently decline order, the cellular immunity persists up to 1 year in anti-SARS-CoV-2 IgG+ recovered individuals. The strength of T cell responses against S1 antigen (in terms of SFC/10^5^ cells) was 8.7 (0–40.2),1.3 (0–5.2), and 1.7 (0–6.7) at 1–2 months, 8–9 months, and 1 year of postrecovery revealing an apparently declining trend with time postinfection.

#### 3.3.3. SARS-CoV-2 S1-Specific T Cell Response in Anti-SARS-CoV-2 IgG− Recovered Individuals

Eleven out of 13 (85%), 1 out of 18 (6%), and 0 out 6 (0%) anti-SARS-CoV-2 IgG− recovered individuals indicated T cell response at 1–2 months, 8–9 months, and 1 year of postrecovery respectively indicating that the cellular immunity persists up to 8–9 months in anti-SARS-CoV-2 IgG− recovered individuals. The strength of T cell responses against S1 antigen (in terms of SFC/10^5^ cells) were 10 (6.15–52.5) and 2.4 (0.57–3.07) at 1–2 months and 8–9 months postrecovery, respectively.

### 3.4. SARS-CoV-2 S1-Specific Cytokines

The levels of proinflammatory IL-1β, IL-5, and IL-6, anti-inflammatory IL-4 and IL-10, cytokines, and chemokines CCL-2 and CCL-3 were significantly low in recovered individuals at R1 and R2 compared to R3. Lower levels of IL-17, TNF-α, chemokine, eotaxin, CCL5, and growth factor GM-CSF were sensed in recovered individuals at R1 compared to R3 only. Lower levels of IFN-γ, FGF basic, G-CSF, and VEGF were detected in recovered individuals at R2 compared to R3 (*p* < 0.008 in each) (Table 5).

### 3.5. Persistence of Neutralization Antibodies Post-SARS-CoV-2 Infection

**Figure 8:**
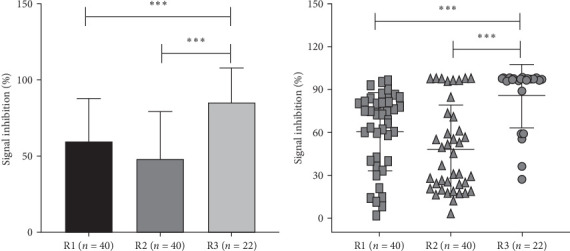
Surrogate neutralization antibody activities against SARS-CoV-2 infection in recovered individuals from SARS-CoV-2 infection. Surrogate neutralization antibody activities against SARS-CoV-2 infection in recovered individuals (1 year postrecovery) from SARS-CoV-2 infection were detected using surrogate virus neutralization (sVNT) assay (cPass, GenScript USA) in plasma samples of recovered individuals R1 (*n* = 40) at 1–2 months, R2 (*n* = 40) at 8–9 months, and R3 (*n* = 22) at 12 months/1 year of postrecovery. The *Y*-axis represents the percentage of signal inhibition (*⁣*^*∗*^*p* value <0.05, *⁣*^*∗∗*^*p* value <0.005, and *⁣*^*∗∗∗*^*p* value <0.0001).

Plasma samples of 34/39 (87.17%, 72.35 [39.89–81.54]) at 1–2 months; 23/40 [57.5%, 61.1 [30.9–98.1]) at 8–9 months; and 21/22 (95.45%, 97.15 [36.21–97.92]) at 1 year had detectable NAbs (Figure 8).

### 3.6. Subgroup Analysis of Recovered Individuals

#### 3.6.1. R1 (Asymptomatic vs. Symptomatic) and (Anti-SARS-CoV-2 IgG+ vs. IgG−)

Flow cytometry analysis displayed significantly higher percentages of CD4+ Th and lower percentages of CD8+ Tc cells in individuals recovered from mild symptomatic infection compared to those recovered from asymptomatic infection (*p* < 0.05 in each). SARS-CoV-2-specific T cell response was detected in 15 of 24 (63%) and 9 of 10 (90%) individuals recovered from asymptomatic and mild symptomatic infections, respectively. Eighteen of 24 (75%) and 15 of 15 (100%) recovered from asymptomatic and mild symptomatic infection had detectable surrogate NAbs. SARS-CoV-2-specific T cell response was noticed in 13 out of 21 (61.90%) and 11 out of 13 (84.61%) anti-SARS-CoV-2 IgG+ and IgG−, respectively. Twenty-five of 25 (100%) anti-SARS-CoV-2 IgG+ had detectable surrogate NAbs (Figure S2A,D).

#### 3.6.2. R2 (Asymptomatic vs. Symptomatic) and (Anti-SARS-CoV-2 IgG+ vs. IgG−)

Flowcytometry analysis displayed significantly higher percentages of CD4+ and CD8+ naïve T cells and significantly lower percentages of CD4+ effector T and CD8+ TEMRA in individuals recovered from symptomatic infection compared to individuals recovered from asymptomatic infection (*p* < 0.05 in each). SARS-CoV-2-specific T cell response was observed in 3 of 21 (14.2%) and 3 of 18 (17%) individuals recovered from asymptomatic and mild symptomatic infections. Plasma samples of 11 of 22 (50%) and 12 of 18 (67%) individuals recovered from asymptomatic and mild symptomatic infections had detectable surrogate NAbs (Figure S2B,E).

The percentages of SARS-CoV-2 S1-stimulated CD8+ naïve cells were significantly higher in anti-SARS-CoV-2 IgG− compared to anti-SARS-CoV-2 IgG+ (*p* < 0.05 in each). SARS-CoV-2-specific T cell response was detected in 5 out of 21 (23.8%) and 1 out of 18 (5.55%) anti-SARS-CoV-2 IgG+ and IgG−. Plasma samples of 19 out of 21 (90%) anti-SARS-CoV-2 IgG+ had detectable surrogate NAbs (Figure S2B,E).

#### 3.6.3. R3 (Asymptomatic vs. Symptomatic) and (Anti-SARS-CoV-2 IgG+ vs. IgG−)

SARS-CoV-2-specific T cell response was detected in 1 out of 12 (8.3%) and 0 out of 10 (0%) individuals recovered from asymptomatic and mild symptomatic infections. Plasma samples of 12 of 12 (100%) and 9 of 10 (90%) individuals recovered from asymptomatic and mild symptomatic infections had detectable surrogate NAbs. SARS-CoV-2-specific T cell response was displayed in 1 of 17 (6%) and 0 of 5 (0%) anti-SARS-CoV-2 IgG+ and IgG−. Plasma samples of 17 of 17 (100%) anti-SARS-CoV-2 IgG+ had detectable surrogate NAbs (Figure S2C,F).

## 4. Discussion

Since the kinetics and longevity of immunological memory in humans following infection or vaccination are generally unpredictable and the immune responses at brief intervals following resolution of infection or postvaccination are not indicative of long-term memory [22, 23], the assessment of multiple parameters of immune responses over a duration of 6 months or more is needed to determine the longevity of immune memory.

Jiang et al. [25] have demonstrated anti-SARS-CoV-2 IgG and NAbs to be detectable and relatively stable at 3–4 months postonset of symptoms in a set of 25 SARS-CoV-2-infected patients. Subsequently, Wajnberget et al. [24] presented that the majority of mild-to-moderate COVID-19-infected individuals showed S protein-specific IgG antibody responses that were stable for approximating 5 months, and its titers correlated with the neutralization of authentic SARS-CoV-2. Studies have correlated the detection of SARS-CoV-2 antibodies up to 6 to 7 months with a significantly reduced risk of reinfection [26, 27]. Deshpande et al.[6] elucidated specific anti-IgG and NAb responses against N and SRBD proteins in the Indian COVID-19 patients up to 8 months. The current data of IgG antibodies and surrogate virus NAbs at 1−2 and 8−9 months postrecovery go hand in hand and showed a declining trend with 65% and 52.5% of individuals having IgG antibodies, while 87.1% and 57.5% of recovered individuals had surrogate virus NAbs, as reported elsewhere [28]. Enhanced IgG and surrogate virus NAb levels in the current 1-year follow-up recovered individuals postremoval of five vaccinated individuals from the group could be attributed to their subclinical reinfection.

Our observation of increased number of IgG anti-SARS-CoV-2 positivity at R3 without any history of reinfection is in line with the published reports indicating that cases of reinfection were more inclined to be asymptomatic or mild when compared to those individuals infected with COVID-19 for the first time [29–32]. Multiple studies described that anti-spike IgG antibody positive healthcare workers had exhibited reduced rates of PCR positivity even after 6 months of follow-up and had overall lower reinfection risk indicating the protective role of IgG antibody [3, 33, 34]. In a similar vein, the detected stronger humoral (IgG and NT responses) and cellular (robust memory B and T cells responses) immune responses in the recovered individuals of our study could be indicative of protective immunity that could have safeguarded the recovered individuals from the circulating VoCs and VoIs, up to post 8–9 months of recovery.

The subgroup analysis data showed detection of surrogate virus NAbs in 75% and 50% of recovered individuals from asymptomatic infection in comparison to 100% and 67% recovered from mild symptomatic infection along with 100% and 90% of anti-SARS-CoV-2 IgG+ at R1 and R2. This elucidated surrogate virus NAbs at R1 and R2 that further established the persistence of NAb responses in Indian COVID-19 patients up to 8–9 months and in abundances in the anti-SARS-CoV-2 IgG+. This information could be instrumental in deciding the time span/gap required for the administration of precautionary booster dose to develop long-term protective immune response/trained immunity against SARS-CoV-2 [35].

T lymphocytes are reported to be the most affected with a decrease in the total number of NK, cytotoxic T cells, and impaired functionality in the early acute stage of COVID-19 infection [36]. A significant decrease in SARS-CoV-2-specific CD4+ T and CD8+ T cells postrecovery have also been demonstrated [15, 37]. The current study indicated lower frequencies of NK and NKT-like cells, higher percentage of CD4+ T post 1–2 months and 8–9 months of recovery, and lower percentages CD8+ T cells at 8–9 months postrecovery, suggesting that complete restoration of peripheral lymphocyte profile may not be a common phenomenon postinfection with SARS-CoV-2. The subanalysis of phenotypic data in asymptomatic and mild symptomatic recovered individuals displayed comparable immune cell profiles except for higher CD4+ Th cells and lower CD8+ Tc cells at R1, higher CD4+ and CD8+ naïve T cells, and lower CD4+ T effector and CD8+ TEMRA cells at R2 in individuals recovered from mild symptomatic infection. It is interesting to note that the immune cell profiles at R1 and R2 were comparable for anti-SARS-CoV-2 IgG+ and IgG−. There is an emerging evidence of potent memory B cell response in COVID-19-recovered individuals [38]. Earlier study reported higher Th memory, Tc memory, and B memory cells in the recovered individuals at 45–60 days postrecovery and suggested that it could be put forward as markers of recovery from mild infection [13]. In addition, memory B cells and T cells confer long-lasting immunity against reinfection. CD4+ helper T cells are important in helping memory B cells and producing NAbs and long-term humoral immunity. Memory cells, detected in 90% of SARS-CoV-2 recovered individuals, have been linked with the prevention of high rates of reinfection [39, 40]. In a parallel line, the detected higher SARS-CoV-2-specific memory B and memory CD4+ T cells in our recovered individuals could also be associated with lower risk of reinfection. The followed-up recovered population of the current study elucidated higher SARS-CoV-2-specific B and memory B cells at 8–9 months which go hand in hand with the report of Gaebler et al. [41], indicating that the identification of memory B cell response to SARS-CoV-2 evolves between 1·3 and 6·2 months after infection, which is aligning with longer-term protection.

The memory immune response in COVID-19 reinfection has not been extensively studied. We have previously reported higher percentages of CD19+ CD27+ B memory cells in the recovered group compared to both asymptomatic, mild symptomatic patient, and uninfected control group [13]. Increased RBD or N-specific memory B cells were detected in 25 patients who were grouped as mild, moderate, or severe from the beginning to 150 days after infection [42]. Winklmeier et al. [43] observed that functional IgG memory B cells persist after 5–8 months in infected patients. Dan et al. [12] demonstrated the existence of circulating memory B cells specific for RBD, N, and S proteins for more than 6 months and up to 8 months PSO in a cohort of 188 cases for long-term studies. On a similar note, our data displayed the presence of circulating anti-SARS-CoV-2 IgG antibodies in 52.5% of recovered individuals and significantly higher percentages of SARS-CoV-2 S1-simulated B and memory B cells and IgG+ B cells at 8–9 months. These observations are significant indicating that in the absence of circulating anti-SARS-CoV-2 IgG antibodies, SARS-CoV-2 S1-specific memory B cells may differentiate into plasma cells upon re-exposure. The aforementioned observation implies that multiple correlates of protective immunity against SARS-CoV-2 are needed for the assessment of the degree of protection conferred by COVID-19 vaccines. This observation also suggests the critical role of memory B cells in immune mechanisms against SARS-CoV-2, specifically toward the detection of previous infection and prediction of long-term protection.

It is a very well-established fact that memory T cells confer protection against previously encountered pathogens [44]. Throughout an individual's lifetime, antigen-experienced T cells accumulate in the memory T cell pool, which serve as a dynamic repository for them. Memory T cells contain distinct populations of naïve, central memory (*T*_CM_), effector memory (*T*_EM_), and terminal effector memory T cells (TEMRA) characterized by their distinct function. Central memory T cells are long-lasting and may be essential for preventing infection [45]. Previously, we have reported significantly higher percentages of CD3+CD4+CD45RO+ Th memory and CD3+CD8+CD45RO+ Tc memory cells in the recovered group compared to both asymptomatic, mild symptomatic patient, and uninfected control group [13]. In the current study, we detected significantly higher CD4+ and CD8+ central memory T cells at 8–9 months postrecovery which is suggestive of long-lasting memory T cell response in recovered individuals.

TEMRA cells are important part of the immune memory, helping to recognize and respond to antigen if re-exposed. However, their persistent presence might also be a double-edged sword, contributing to prolonged symptoms [46]. In the context of COVID-19, the role of TEMRA cells is not fully understood. Saihati et al. [47] demonstrated a significant elevation in CD8+ TEMRA level in SARS-CoV-2-infected patients compared to control. In patients with both severe and critical COVID-19 illness, Anft et al. have shown reduced frequencies of terminally differentiated T cell subsets [48]. Jung et al.'s [49] findings also revealed that COVID-19 convalescent patients had reduced expression of TEMRA cells for a period of 10 months. In our study, we detected significantly lower levels of CD8+ TEMRA in recovered individuals at 8–9 months of recovery, suggesting that the immune system may require some time to return to normal following COVID-19 infection. CD4+ TEMRA cells are associated with protective immunity against pathogens, including dengue virus [50]. In a similar line, the detected significantly higher percentages of CD4+ TEMRA cells in our study are indicative of protective immunity in the recovered individuals at 8–9 months postrecovery. Collectively, these observations might help in assessing the risk of reinfection or the severity of future SARS-CoV-2 infections. More studies are needed to fully understand their role and implications in the context of COVID-19 recovery and reinfection.

Central memory is more beneficial in diseases with long incubation periods, whereas effector memory is essential for safeguarding against the most infections [17]. Detected lower percentages of phenotypic CD4+ and CD8+ effector T memory cells at R2 could be suggestive of either their natural decline or exhaustion due to prolonged immune activation during the course of the infection. However, it is difficult to comment as T cells exhaustion markers are not studied in the current study population. This highlights the importance of monitoring immune memory and potentially considering booster vaccinations to maintain robust immunity. Overall, based on the observations, it seems that the development of B cell memory to SARS-CoV-2 was robust and is also likely long-lasting. These observations indicate that memory T and B cells persist up to 8–9 months postinfection/recovery, in agreement with the status of cellular and humoral immunity. Higher production of IFN-γ/CD107a by CD8+T cells and NK cells in recovered individuals 8–9 months postrecovery could probably be associated with a higher number of anti-S-RBD-specific cytotoxic T and NK cells and suggestive of the robust anti-spike T cell response in naturally recovered individuals. The presence of memory T and B cells with the functional ability to generate antibodies immediately upon reintroduction of a pathogen may act as a correlate of protection against SARS-CoV-2 infection by providing continuous protective immunity. Subgroup analysis data indicated that both at R2 and R3, SARS-CoV-2-specific immune cell profiles, and their functionality (in terms of CD 107a/IFN-γ expressions) were comparable irrespective of their anti-SARS-CoV-2 antibody status/the clinical presentation of COVID-19 at the time of infection. Chia et al. [51] described the maintenance of substantial specific T cells at 6 months postsymptom onset and multispecific T cell response reactive to NP, M, and S peptides in randomly selected 23 COVID-19 recovered individuals. Patients from the antibody negative group also showed sustained T cell immunity 6 months after initial infection [51]. In a similar line, Peluso et al. [52] reported variability in T cell responses over the long term for up to 9 months in the individuals recovering from SARS-CoV-2 infection. The subgroup analysis data demonstrated detection of SARS-CoV-2-specific T cell response in 61.9%, 23.8%, and 6% in anti-SARS-CoV-2 IgG+ in comparison to 84.6%, 5.5%, and 0% in anti-SARS-CoV-2 IgG− recovered individuals which clearly represented persistence of effector immunity up to 8–9 months in the recovered individuals irrespective of IgG status. The sequential data demonstrated persistence of SARS-CoV-2-specific T cell response in a single recovered individual (1 of 8, 12.5%) throughout the assessed time points up to 1 year postrecovery, hereby providing the evidence of cellular immune response to SARS-CoV-2.

In a set of individuals at 1–2 months postrecovery, lower plasma IL-6 and IL-1β levels have previously reported as markers of recovery [21], and the current study indicates that this statement is valid up to 8–9 months postrecovery. This may speculate a long-lasting cytokine signature in naturally recovered individuals at 1–2 months and 8–9 months postrecovery. Detection of higher level of IFN-γ in recovered individuals at 1 year might be due to subclinical reinfection. Multiple IgG+ ELISAs/PCR tests performed at regular intervals prior to inclusion in the study as controls would leave no room for individuals with prior asymptomatic infection who have become negative for IgG anti-SARS-CoV-2 antibody. However, the included IgG anti-SARS-CoV-2 antibody negatives as controls did not have any SARS-CoV-2-specific T cell response, SARS-CoV-2-specific immune cells, or T/NK cell effector functionality indicating that they were truly SARS-CoV-2 naïve. The theme of the current study was to assess the durability of immune response postrecovery from SARS-CoV-2, and the focus was on the recovered individuals at different time points postrecovery, and hence, the crucial comparisons were among these recovered categories only.

Though the induction of sterilizing immunity via high titers of NAbs is a best-case scenario, viruses that infect via the mucus membranes of the nose and throat typically do not induce sterilizing immunity. Immune memory responses from either primaryinfection or vaccination are also the sources of protective immunity against a subsequent infection [13, 22, 53–55].

## 5. Conclusion

Here, following 40 COVID-19 recovered individuals at 1–2 months, 8–9 months, and 1 year postrecovery, higher SARS-CoV-2-specific T cells, B, memory B, and plasma cells that persisted up to 8–9 months postprimary infection were detected even after decline in IgG and NAbs. The recovered individuals at 8–9 months exhibited expressions of IFN-γ and CD107a by NK and CD8 T cells indicating SARS-CoV-2-specific effector functions. We also detected SARS-CoV-2-specific T cells at 8–9 month that were capable of effector function in secreting IFN-γ during the antigen recall response by ELISPOT. These findings of durability of T cell immunity in recovered individuals are encouraging and may assure protection via T cell-governed effector mechanisms.

## Figures and Tables

**Table 1:** Characteristics of recovered individuals from SARS-CoV-2 infection and healthy/uninfected controls.

	R1 (recovered at 1–2 months postrecovery)	R2 (recovered at 8–9 months post recovery)	R3 (recovered at 1 year postrecovery)	HC (healthy/uninfected controls)
Study population	*n* = 40	*n* = 40	*n* = 27*⁣*^*∗*^	*n* = 56

Sex ratio (male:female)	1.35	1.22	1.45	1.54

Age (years): median (range)	38 (18–70)	36 (19–64)	38 (19–64)	27 (18–47)

Recovered from asymptomatic and symptomatic infection	Asymptomatic: 24 (60%) Mild symptomatic: 16 (40%)	Asymptomatic: 22 (55%) Mild symptomatic: 18 (45%)	Asymptomatic: 14 (51.8%) Mild symptomatic: 13 (48.14%)	NA

Anti-SARS-CoV-2 IgG status	Positive: 26 (65%) Negative: 14 (35%)	Positive: 21 (52.5%) Negative: 19 (47.5%)	Positive: 21 (77.77%) Negative: 6 (22.22%)	Positive: 0 Negative: 56

Reinfection from SARS-CoV-2	No	No	No	NA

Vaccination for COVID-19	No	No	#5 out of 27 (first dose)	No

Abbreviations: COVID-19, coronavirus disease-19; NA, not applicable; SARS-CoV-2, severe acute respiratory syndrome coronavirus 2.

*⁣* ^*∗*^5/27 at R3 were reported to be vaccinated with 1st dose of SARS-CoV-2, hence removed from further analysis.

**Table 2:** Percentages of NK/NKT-like, T, and B cells and their subsets in SARS-CoV-2-recovered individuals and healthy/uninfected controls.

Cell types	R1 (recovered at 1–2 months post recovery, *n* = 40)	*p* value^a^	R2 (recovered at 8–9 months post recovery, *n* = 40)	*p* value^b^	R3 (recovered at 1 year postrecovery, *n* = 22)	*p* value^c^	HC (healthy/uninfected controls, *n* = 56)	*p* value^d^	*p* value^e^	*p* value^f^
NK and NKT cells										
NK cells	2.2 (1.3–3.27)	<0.0001	16.5 (9.25–26.9)	ns	12.4 (8.9–17.8)	ns	16.7 (12.20–22.05)	<0.0001	<0.0001	ns
NKT cells	4.2 (2.2–5.85)	ns	5.8 (4.0–7.47)	ns	5.8 (3.75–9.55)	ns	4.9 (3.15–7.7)	0.006	0.027	ns
T and memory T cell profile										
CD4+ Th cells	71.4 (63.3–83.2)	0.034	73.3 (62.78–81.65)	0.03	53.7 (46.63–71.18)	ns	65.6 (51.2–76.8)	ns	0.0001	0.0003
CD8+ Tc cells	16.4 (9.1–30.53)	ns	8.7 (3.02–17.48)	ns	22.2 (13.38–30.95)	0.03	13.5 (6.4–24.2)	0.0014	ns	<0.0001
CD4+ naïve T cells	NA		41.1 (24.23–53.9)	0.02	9.7 (4.65–26)	ns	22.4 (3.9–47.3)	NA		0.0001
CD4+ *T*_CM_ cells			34.2 (28–41.53)	0.04	15.7 (3.175–35.85)	ns	24.4 (8.6–40.1)			0.0006
CD4+ TEMRA cells			19.4 (11.98–24.9)	ns	38.4 (27.03–52.68)	ns	26.6 (14.3–38.2)			0.0001
CD4+ effector T cells			2.3 (1.3–6.07)	0.01	14.9 (4.625–36.48)	0.025 ns	2.7 (0.7–40.8)			0.0001
CD8+ naïve T cells			20 (13.3–44.5)	ns	2.4 (0.55–24.65)	ns	15.1 (3.8–25.8)			0.034
CD8+ *T*_CM_ cells			3.95 (1.7–7.67)	ns	0.8 (0.2–4.3)	ns	1.9 (0.92–7.95			0.04
CD8+ TEMRA cells			11 (5.97–17.43)	ns	36.8 (19.95–50)	0.017	17.3 (11.8–32.8)			ns
CD8+ effector T cells			56.35 (34.95–69.35)	0.028	48.4 (35.85–59.28)	0.00010	45.6 (26.5–64.8)			0.013 ns
B and memory B cell profile										
B cells	4.5 (2.4–5.8)	<0.0001	9.4 (6.2–14.5)	ns	9.85 (6.5–16.9)	ns	9.9 (7.5–12.4)	<0.0001	<0.0001	ns
Memory B cells	17.2 (12.8–24.5)	ns	24.7 (16.5–30.3)	ns	16.7 (13.9–21.43)	0.05	19.3 (17.5–25.4)	0.007	ns	0.004
IgG+ B cells	NA	ns	8.7 (5.65–11.3)	ns	17.7 (8.3–33.9)	ns	8.2 (5.9–12.2)	NA	NA	ns
IgG+ memory B cells		NA	22.8 (19.6–30.2)	ns	21.0 (2.6–40.7)	ns	20.8 (15.7–24.9			ns

*Note:* Percentages of immune cells are represented as median (IQR), *p* values. A *p* value <0.05 is considered significant. R1, recovered individuals at 1–2 months postrecovery; R2, recovered individuals at 8–9 months postrecovery; R3, recovered individuals at 1 year postrecovery.

Abbreviations: NA, not available; NK, natural killer; NKT, natural killer-like T; ns, nonsignificant; SARS-CoV-2, severe acute respiratory syndrome coronavirus 2.

^a^R1 versus uninfected controls.

^b^R2 versus uninfected controls.

^c^R3 versus uninfected controls.

^d^R1 versus R2.

^e^R1 versus R3.

^f^R2 versus R3.

**Table 3:** Percentages of SARS-CoV-2 S1-simulated B, memory B cell subsets, and CD4+ and CD8+ memory T cell subsets in SARS-CoV-2-recovered individuals and healthy/uninfected controls.

Cell types	R2 (recovered at 8–9 months postrecovery, *n* = 40)	*p* value^a^	R3 (recovered at 1 year postrecovery, *n* = 22)	*p* value^b^	*p* value^c^	HC (healthy/uninfected controls, *n* = 17)
B and memory B cell profile						
B cells	0.1 (0–0.9)	ns	0.1 (0–1.1)	0.05	ns	0 (0–0.10)
Memory B cells	0.2 (0–1.2)	ns	0 (0–1.1)	0.05	ns	0 (0–0)
IgG+ B cells	0.2 (0–1.2)	ns	1 (0–9.0)	0.05	0.05	0 (0–0.07)
IgG+ memory B cells	0.6 (0–2.3)	ns	0.5 (0–3.2)	ns	ns	ns
T and memory T cell profile						
CD4 Th cells	0.2 (0–3.0)	ns	0.1 (0–4.9)	NA		
CD8+ Tc cells	0 (0–1.0)	ns	0 (0–2.6)			
CD4+ naïve T cells	0 (0–1.3)	ns	0 (0–4.8)			
CD4+ *T*_CM_ cells	0.7 (0–2.5)	ns	0 (0–0.8)			
CD4+ TEMRA cells	0.3 (0–2.6)	ns	0 (0–0.1)			
CD4+ effector T cells	0 (0–1.2)	ns	0.8 (0–5.8)			
CD8+ naïve T cells	0 (0–1.0)	ns	0 (0–0.1)			
CD8+ *T*_CM_ cells	0 (0–0.5)	ns	0 (0–0.5)			
CD8+ TEMRA cells	0 (0–1.7)	ns	0.8 (0–5.7)			
CD8+ effector T cells	0 (0–1.8)	ns	1.3 (0–3.5)			

*Note:* Percentages of immune cells are represented as median (IQR); *p* values. A *p* value <0.05 is considered significant.

Abbreviations: NA, not available; ns, nonsignificant; SARS-CoV-2, severe acute respiratory syndrome coronavirus 2.

^a^R2 versus R3.

^b^R2 versus uninfected controls.

^c^R3 versus uninfected controls.

**Table 4:** T and NK cell effector response in SARS-CoV-2-recovered individuals post-SARS-CoV-2 infection at R2 and R3.

Cell types	R2 (recovered at 8–9 months postrecovery, *n* = 24)	R2 vs. R3 (*p* value)	R3 (recovered at 1 year postrecovery, *n* = 13)
CD107a+ NK cells	2.85 (1.13–5.83)	0.05	1.3 (0.30–2.15)
IFN-γ+ NK cells	0.95 (0.35–3.15)	ns	0.5 (0.15–2.25)
CD107a+ CD4+ T cells	0.7 (0.40–1.95)	ns	0.8 (0.30–2.40)
IFN-γ+ CD4+ T cells	0.6 (0.23–1.50)	0.05	0.2 (0.00–0.45)
CD107a+ CD8+ T cells	2.7 (1.40–4.50)	0.001	0.8 (0.05–1.40)
IFN-γ+ CD8+ T cells	0.9 (0.50–2.30)	0.005	0 (0.00–0.65)

*Note*: Percentages of immune cells are represented as median (IQR), *p* values. R2 versus R3; *p* value <0.05 is considered significant.

Abbreviations: NK, natural killer; ns, nonsignificant; SARS-CoV-2, severe acute respiratory syndrome coronavirus 2.

**Table 5:** Levels of SARS-CoV-2-specific cytokines/chemokines in recovered individuals post 1–2 months, 8–9 months, and 1 year of recovery from SARS-CoV-2 infection and in uninfected controls.

Analytes	HC (*n* = 7)	*p* value^a^	R1 (*n* = 14)	*p* value^b^		R2 (*n* = 14)	*p* value^c^	R3 (*n* = 17)	*p* value^d^	*p* value^e^	*p* value^f^
Proinflammatory cytokines											
IL-1β	8.75 (3.72–407.8)	0.005	0 (0–0.81)	ns	0.82 (0–2.79)		0.008	713.5 (433.4–1150)	ns	<0.001	<0.001
IL-5	12.04 (0–17.69)	ns	8.2 (0–18.04)	ns	13.95 (0–40.52)		ns	79.12 (41.9–115.4)	ns	0.001	0.007
IL-6	73.32 (0–95.72)	ns	4.13 (0–96.11)	ns	77.81 (0–289.5)		ns	8831 (2374–13106)	ns	0.002	0.004
IL-7	0 (0–1.56)	ns	0 (0–4.82)	ns	0 (0–8.24)		ns	0 (0–5.69)	ns	ns	ns
IL-9	5.51 (0–26)	ns	6.06 (0–12.99)	ns	0.37 (0–36.78)		ns	21.71 (0–94.91)	ns	ns	ns
IL-15	61.92 (0–86.97)	ns	0 (0–82.22)	ns	0 (0–16.88)		ns	0 (0–132.1)	ns	ns	ns
IL-17	11.25 (0–24.22)	ns	0 (0–0.15)	ns	1.63 (0–9.83)		ns	10.77 (0–31.4)	ns	0.004	ns
TNF-α	47.76 (0–5010)	ns	0 (0–41.09)	ns	15.87 (4.46–61.49)		ns	179.1 (0–1570)	ns	ns	ns
Anti-inflammatory cytokines											
IL-1RA	599.5 (0–1529)	ns	37.65 (0–318)	ns	23.94 (0–189.3)		ns	6424 (0–9720)	ns	ns	ns
IL-4	0.76 (0–0.88)	ns	0.03 (0–0.39)	ns	0.115 (0–0.47)		ns	3.41 (0.65–5.93)	ns	0.001	0.001
IL-10	0 (0–3.38)	ns	0.62 (0–2.61)	ns	0.65 (0–1.54)		ns	53.9 (16.37–139)	ns	0.001	0.001
IL-13	0 (0–0.56)	ns	0 (0–0.85)	ns	0.4 (0–2.25)		ns	0.7 (0–2.84)	ns	ns	ns
TH1 cytokines											
IL-2	4.69 (0–18.59)	ns	1.28 (0–6.63)	ns	2.53 (0–10.35)		ns	29.6 (0–60.55)	ns	ns	ns
IFN-γ	1.2 (0–85.36)	ns	4.03 (0–36)	ns	0 (0–9.90)		ns	53.35 (0–81.64)	ns	ns	0.003
IL-12 (p70)	0 (0–3.08)	ns	0 (0–0.73)	ns	3.09 (0–4.55)		ns	1.52 (0–4.31)	ns	ns	ns
Chemokines											
Eotaxin	0.19 (0–0.92)	ns	0.05 (0–0.32)	ns	0.14 (0–1.01)		ns	4.56 (0.23–7.44)	ns	0.002	ns
CCL-2	4.91 (0–212.3)	ns	0 (0–0.62)	ns	0 (0–25.88)		ns	149.3 (0–720.4)	ns	0.001	0.003
CCL-3	12.01 (5.15–33.82)	ns	0.14 (0–2.54)	ns	5.21 (0–44.46)		ns	1336 (51.58–2912)	ns	0.002	0.003
CCL-4	52.4 (0–468.2)	ns	6.6 (0–25.89)	ns	67.06 (0–189.2)		ns	527.7 (0–2513)	ns	ns	ns
CCL-5	120.3 (0–1416)	ns	0 (0–49.81)	ns	217.6 (0–618.5)		ns	546.4 (0–7798)	ns	0.006	ns
IL-8	217.6 (0–5299)	ns	654.5 (05843)	ns	418.4 (0–7604)		ns	666.3 (0–4717)	ns	ns	ns
CXCL-10	4.16 (0–405)	ns	2225(114.5–5696)	ns	38.63 (0–1757)		ns	37.8 (0–677.6)	ns	ns	ns
Growth factor											
FGF basic	14.96 (0–18.92)	ns	0(0–6.58)	ns	0 (0–2.03)		ns	67.33 (0–75.26)	ns	ns	0.005
G-CSF	105.8 (0–856.7)	ns	6.21 (0–57.65)	ns	0 (0–132.6)		ns	1943 (0–7900)	ns	ns	0.002
GM-CSF	1.76 (0–77.09)	ns	0 (0–1.41)	ns	1.04 (0–3.19)		ns	9.96 (0.36–227.7)	ns	0.004	ns
VEGF	15.16 (0–93.18)	ns	14.38 (0–95.5)	ns	14.47 (0–39.18)		ns	194 (12.33–431.5)	ns	ns	0.007
PDGF-BB	0 (0–67.3)	ns	13.88 (0–154.6)	ns	7.2 (0–18.12)		ns	314 (0–593.1)	ns	ns	ns

*Note*: The Bonferroni's correction was applied to consider multiple comparisons for each analyte. Thus, the conventional cutoff for *p* value (0.05) was lowered down by dividing it by the number of comparisons made for each analyte and considered *p* value as 0.008 for cytokine comparison.

Abbreviations: FGF, fibroblast growth factor; HC, healthy/uninfected controls; ns, nonsignificant; SARS-CoV-2, severe acute respiratory syndrome coronavirus 2.

^a^Recovered at 1–2 months postrecovery versus healthy/uninfected controls.

^b^Recovered at 8–9 months postrecovery versus healthy/uninfected controls.

^c^Recovered at 12 months/1 year months postrecovery versus healthy/uninfected controls.

^d^Recovered at 1–2 months postrecovery versus recovered at 8–9 months postrecovery.

^e^Recovered at 1–2 months postrecovery versus recovered at 12 months/1 year postrecovery.

^f^Recovered at 8–9 months postrecovery versus recovered at 12 months/1 year postrecovery.

*⁣* ^*∗*^Values of cytokines and chemokines are represented as median (IQR), *p* values.

## Data Availability

The data that support the findings of this study are available on request from the corresponding author. The data are not publicly available due to ethical restrictions.

## Supporting Information

Supporting Information Supporting figures were provided.

## References

[1] Barouch D. H. (2022). Covid-19 Vaccines—Immunity, Variants, Boosters. *New England Journal of Medicine*.

[2] Yadav P. D., Sahay R. R., Salwe S. (2023). Broadly Reactive SARS-CoV-2-Specific T-Cell Response and Participation of Memory B and T Cells in Patients With Omicron COVID-19 Infection. *Journal of Immunology Research*.

[3] Lumley S. F., O’Donnell D., Stoesser N. E. (2021). Antibody Status and Incidence of SARS-CoV-2 Infection in Health Care Workers. *New England Journal of Medicine*.

[4] Galipeau Y., Greig M., Liu G., Driedger M., Langlois M. A. (2020). Humoral Responses and Serological Assays in SARS-CoV-2 Infections. *Frontiers in Immunology*.

[5] Wölfel R., Corman V. M., Guggemos W. (2020). Virological Assessment of Hospitalized Patients With COVID-2019. *Nature*.

[6] Deshpande G. R., Kaduskar O., Deshpande K. (2021). Longitudinal Clinico-Serological Analysis of Anti-Nucleocapsid and Anti-Receptor Binding Domain of Spike Protein Antibodies Against SARS-CoV-2. *International Journal of Infectious Diseases*.

[7] Blanco-Melo D., Nilsson-Payant B. E., Liu W. C. (2020). SARS-CoV-2 Launches a Unique Transcriptional Signature From in Vitro, Ex Vivo, and in Vivo Systems.

[8] Callow K. A., Parry H. F., Sergeant M., Tyrrell D. A. J. (1990). The Time Course of the Immune Response to Experimental Coronavirus Infection of Man. *Epidemiology and Infection*.

[9] Cao X. (2020). COVID-19: Immunopathology and Its Implications for Therapy. *Nature Reviews Immunology*.

[10] Tan Y., Liu F., Xu X. (2020). Durability of Neutralizing Antibodies and T-Cell Response Post SARS-CoV-2 Infection. *Frontiers of Medicine*.

[11] Quast I., Tarlinton D. (2021). B Cell Memory: Understanding COVID-19. *Immunity*.

[12] Dan J. M., Mateus J., Kato Y. (2021). Immunological Memory to SARS-CoV-2 Assessed for up to 8 Months After Infection. *Science*.

[13] Tripathy A. S., Trimbake D., Suryawanshi P. V. (2022). Peripheral Lymphocyte Subset Alteration in Patients With COVID-19 Having Differential Clinical Manifestations. *Indian Journal of Medical Research*.

[14] Pulendran B., Oh J. Z., Nakaya H. I., Ravindran R., Kazmin D. A. (2013). Immunity to Viruses: Learning From Successful Human Vaccines. *Immunological Reviews*.

[15] Kang C. K., Kim M., Lee S. (2021). Longitudinal Analysis of Human Memory T-Cell Response According to the Severity of Illness up to 8 Months After Severe Acute Respiratory Syndrome Coronavirus 2 Infection. *The Journal of Infectious Diseases*.

[16] Thanapati S., Das R., Tripathy A. S. (2015). Phenotypic and Functional Analyses of NK and NKT-Like Populations During the Early Stages of Chikungunya Infection. *Frontiers in Microbiology*.

[17] Kulkarni S. P., Sharma M., Tripathy A. S. (2019). Antibody and Memory B Cell Responses in Hepatitis E Recovered Individuals, 1–30 Years Post Hepatitis E Virus Infection. *Scientific Reports*.

[18] Thanapati S., Ganu M. A., Tripathy A. S., Roques P. (2017). Differential Inhibitory and Activating NK Cell Receptor Levels and NK/NKT-Like Cell Functionality in Chronic and Recovered Stages of Chikungunya. *PLOS ONE*.

[19] Tripathy A. S., Das R., Chadha M. S., Arankalle V. A. (2011). Epidemic of Hepatitis B With High Mortality in India: Association of Fulminant Disease With Lack of CCL4 and Natural Killer T Cells. *Journal of Viral Hepatitis*.

[20] Rathod S. B., Tripathy A. S. (2014). Hepatitis E rORF2p Stimulated and Unstimulated Peripheral Expression Profiling in Patients With Self-Limiting Hepatitis E Infection. *Journal of Immunology Research*.

[21] Tripathy A. S., Vishwakarma S., Trimbake D. (2021). IL-1*β*, and IL-6: Biomarkers of SARS-CoV-2 Infection. *Archives of Virology*.

[22] Sallusto F., Lanzavecchia A., Araki K., Ahmed R. (2010). From Vaccines to Memory and Back. *Immunity*.

[23] Crotty S., Ahmed R. (2004). Immunological Memory in Humans. *Seminars in Immunology*.

[24] Wajnberg A., Amanat F., Firpo A. (2020). Robust Neutralizing Antibodies to SARS-CoV-2 Infection Persist for Months. *Science*.

[25] Jiang X. L., Wang G. L., Zhao X. N. (2021). Lasting Antibody and T Cell Responses to SARS-CoV-2 in COVID-19 Patients Three Months After Infection. *Nature Communications*.

[26] Clarke C. L., Prendecki M., Dhutia A. (2021). Longevity of SARS-CoV-2 Immune Responses in Hemodialysis Patients and Protection Against Reinfection. *Kidney International*.

[27] Ortega N., Ribes M., Vidal M. (2021). Seven-Month Kinetics of SARS-CoV-2 Antibodies and Role of Pre-Existing Antibodies to Human Coronaviruses. *Nature Communications*.

[28] Feng C., Shi J., Fan Q. (2021). Protective Humoral and Cellular Immune Responses to SARS-CoV-2 Persist up to 1 Year After Recovery. *Nature Communications*.

[29] Hall V. J., Foulkes S., Charlett A. (2021). SARS-CoV-2 Infection Rates of Antibody-Positive Compared With Antibody-Negative Health-Care Workers in England: A Large, Multicentre, Prospective Cohort Study (SIREN). *The Lancet*.

[30] Letizia A. G., Ge Y., Vangeti S. (2021). SARS-CoV-2 Seropositivity and Subsequent Infection Risk in Healthy Young Adults: A Prospective Cohort Study. *The Lancet Respiratory Medicine*.

[31] Young K., Baumeister A., Corrin T., Waddell V. (2022). Rapid Review on Protective Immunity Post Infection With SARS-CoV-2: Update 3—Canada.ca.

[32] Chansaenroj J., Yorsaeng R., Posuwan N. (2021). Long-Term Specific IgG Response to SARS-CoV-2 Nucleocapsid Protein in Recovered COVID-19 Patients. *Scientific Reports*.

[33] Wilkins J. T., Hirschhorn L. R., Gray E. L. (2022). Serologic Status and SARS-CoV-2 Infection Over 6 Months of Follow up in Healthcare Workers in Chicago: A Cohort Study. *Infection Control & Hospital Epidemiology*.

[34] Iversen K., Kristensen J. H., Hasselbalch R. B. (2022). Seroprevalence of SARS-CoV-2 Antibodies and Reduced Risk of Reinfection Through 6 Months: A Danish Observational Cohort Study of 44 000 Healthcare Workers. *Clinical Microbiology and Infection*.

[35] Ahluwalia P., Vaibhav K., Ahluwalia M. (2021). Infection and Immune Memory: Variables in Robust Protection by Vaccines Against SARS-CoV-2. *Frontiers in Immunology*.

[36] Le Bert N., Tan A. T., Kunasegaran K. (2020). SARS-CoV-2-Specific T Cell Immunity in Cases of COVID-19 and SARS, and Uninfected Controls. *Nature*.

[37] Shrotri M., van Schalkwyk M. C., Post N. (2021). T Cell Response to SARS-CoV-2 Infection in Humans: A Systematic Review. *PLOS ONE*.

[38] Sokal A., Barba-Spaeth G., Fernández I. (2021). MRNA Vaccination of Naive and COVID-19-Recovered Individuals Elicits Potent Memory B Cells That Recognize SARS-CoV-2 Variants. *Immunity*.

[39] Syeda M. Z., Hong T., Huang C., Huang W., Mu Q. (2024). B Cell Memory: From Generation to Reactivation: A Multipronged Defense Wall Against Pathogens. *Cell Death Discovery*.

[40] Hermens J. M., Kesmir C. (2023). Role of T Cells in Severe COVID-19 Disease, Protection, and Long Term Immunity. *Immunogenetics*.

[41] Gaebler C., Wang Z., Lorenzi J. C. (2021). Evolution of Antibody Immunity to SARS-CoV-2. *Nature*.

[42] Röltgen K., Boyd S. D. (2021). Antibody and B Cell Responses to SARS-CoV-2 Infection and Vaccination. *Cell Host & Microbe*.

[43] Winklmeier S., Eisenhut K., Taskin D. (2022). Persistence of Functional Memory B Cells Recognizing SARS-CoV-2 Variants Despite Loss of Specific IgG. *Iscience*.

[44] Omilusik K. D., Goldrath A. W. (2017). The Origins of Memory T Cells. *Nature*.

[45] Sallusto F., Geginat J., Lanzavecchia A. (2004). Central Memory and Effector Memory T Cell Subsets: Function, Generation, and Maintenance. *Annual Review of Immunology*.

[46] Guo L., Liu X., Su X. (2023). The Role of TEMRA Cell-Mediated Immune Senescence in the Development and Treatment of HIV Disease. *Frontiers in Immunology*.

[47] Al Saihati H. A., Hussein H. A., Thabet A. A. (2023). Memory T Cells Discrepancies in COVID-19 Patients. *Microorganisms*.

[48] Anft M., Paniskaki K., Blazquez-Navarro A. (2020). COVID-19 Progression Is Potentially Driven by T Cell Immunopathogenesis.

[49] Jung J. H., Rha M. S., Sa M. (2021). SARS-CoV-2-Specific T Cell Memory Is Sustained in COVID-19 Convalescent Patients for 10 Months With Successful Development of Stem Cell-Like Memory T Cells. *Nature Communications*.

[50] Tian Y., Babor M., Lane J. (2017). Unique Phenotypes and Clonal Expansions of Human CD4 Effector Memory T Cells Re-Expressing CD45RA. *Nature Communications*.

[51] Chia W. N., Zhu F., Ong S. W. (2021). Dynamics of SARS-CoV-2 Neutralising Antibody Responses and Duration of Immunity: A Longitudinal Study. *The Lancet Microbe*.

[52] Peluso M. J., Deitchman A. N., Torres L. (2021). Long-Term SARS-CoV-2-Specific Immune and Inflammatory Responses in Individuals Recovering From COVID-19 With and Without Post-Acute Symptoms. *Cell Reports*.

[53] Orenstein W. A., Ahmed R. (2017). Simply Put: Vaccination Saves Lives. *Proceedings of the National Academy of Sciences*.

[54] Piot P., Larson H. J., O’Brien K. L. (2019). Immunization: Vital Progress, Unfinished Agenda. *Nature*.

[55] Tripathy A. S., Trimbake D., Singh D. (2024). Abstracts of the Papers Presented in the International Conference of Indian Virological Society, VIROCON. 2023 on, Advancements in Global Virus Research Towards One Health, Held During 01–03 December, 2023 at ICAR—National Research Centre for Banana, Tiruchirappalli, Tamil Nadu, India. *VirusDisease*.

