# Supplementary material for: Durability of Functional SARS-CoV-2-Specific Immunological Memory and T Cell Response up to 8–9 Months Postrecovery From COVID-19
Source: J Immunol Res. 2025 Feb 10;2025:9743866. doi: 10.1155/jimr/9743866 (PMC11832264; doi:10.1155/jimr/9743866)
Supplement: Supporting Information — Supporting figures were provided. [file 9743866.f1.pptx]

## Slide 1
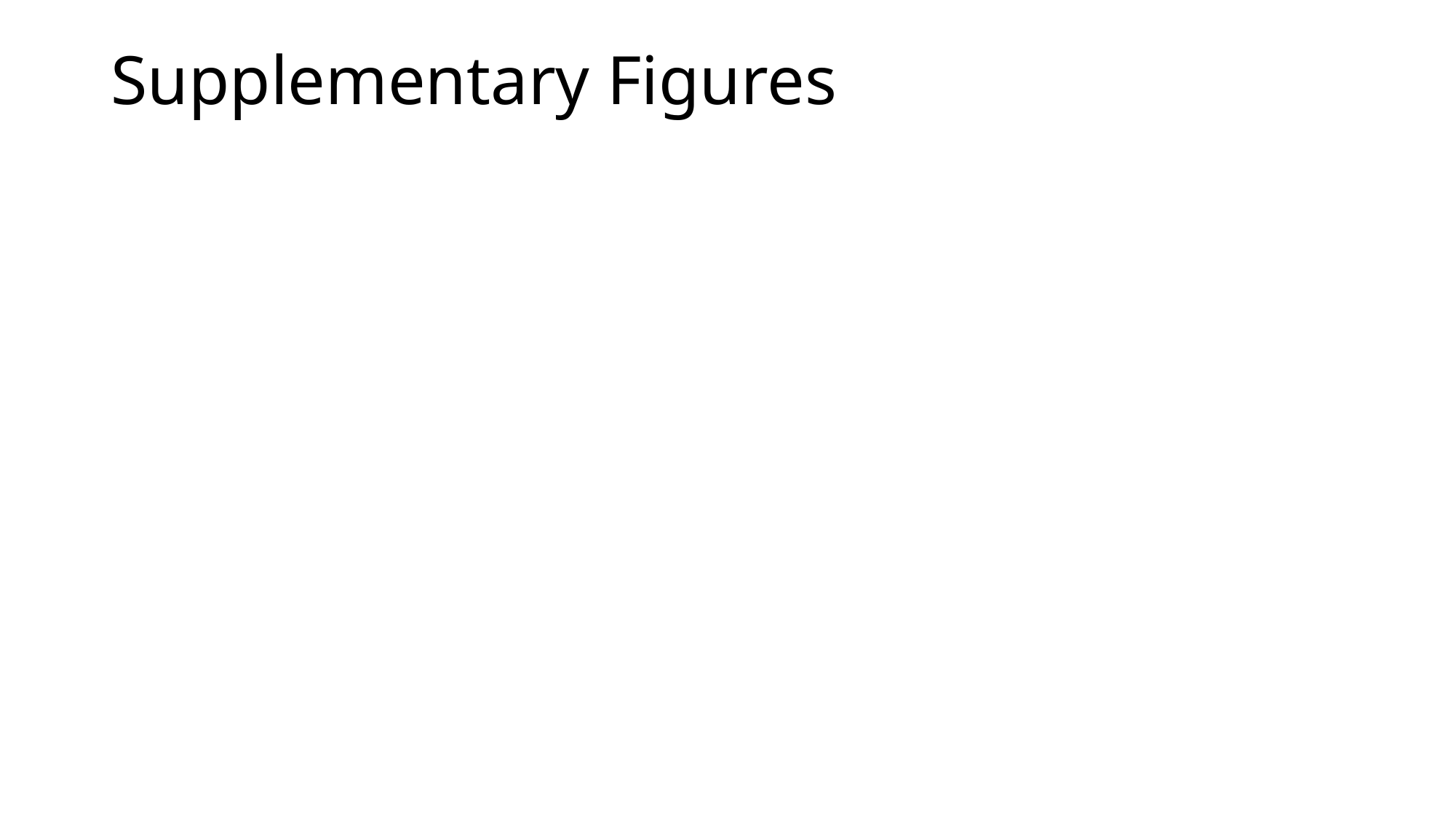

# Supplementary Figures

## Slide 2
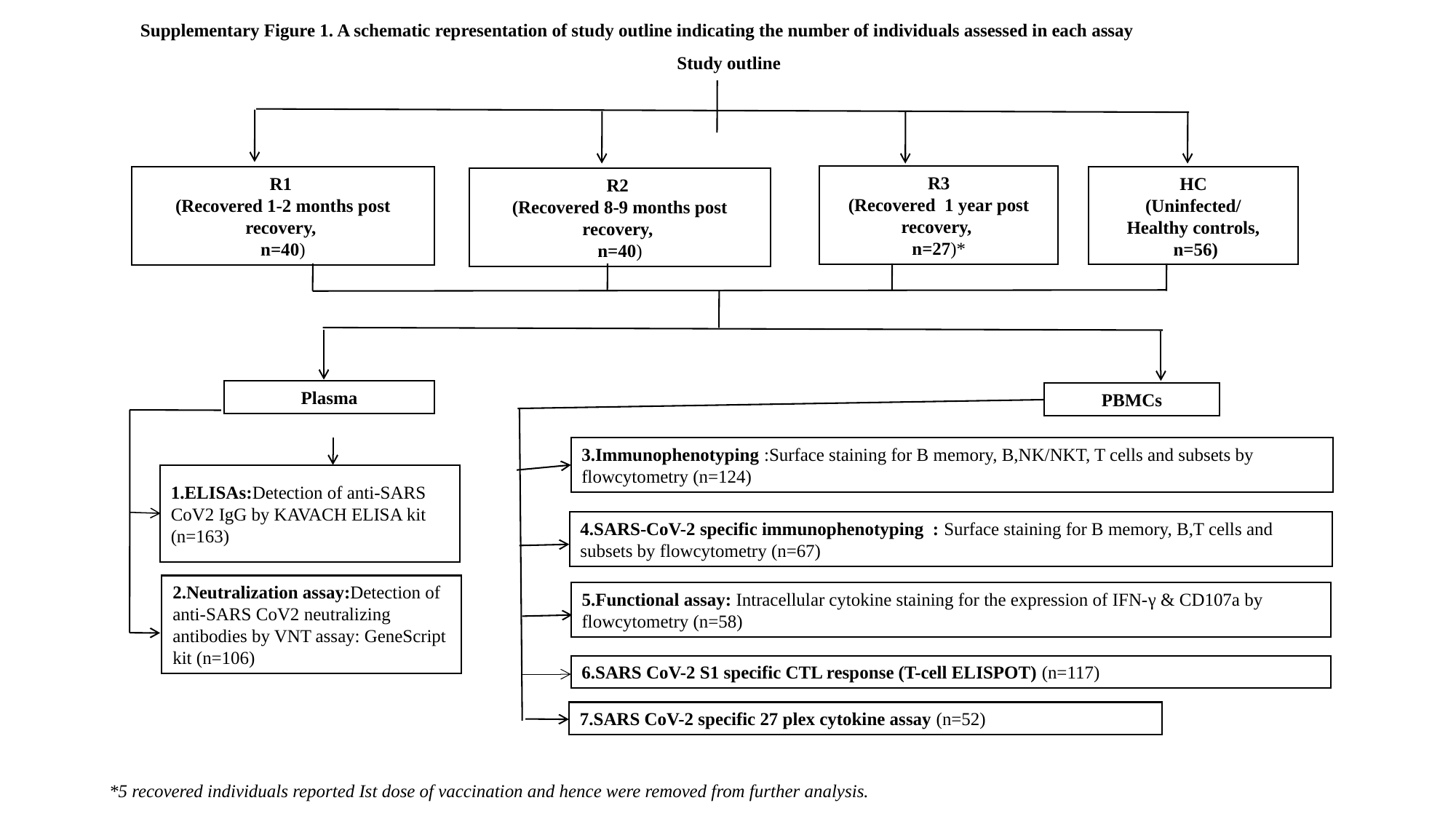

Supplementary Figure 1. A schematic representation of study outline indicating the number of individuals assessed in each assay
Study outline
HC
(Uninfected/
Healthy controls,
 n=56)
R1
(Recovered 1-2 months post recovery,
n=40)
3.Immunophenotyping :Surface staining for B memory, B,NK/NKT, T cells and subsets by flowcytometry (n=124)
6.SARS CoV-2 S1 specific CTL response (T-cell ELISPOT) (n=117)
R2
(Recovered 8-9 months post recovery,
n=40)
Plasma
PBMCs
R3
(Recovered 1 year post recovery,
n=27)*
1.ELISAs:Detection of anti-SARS CoV2 IgG by KAVACH ELISA kit (n=163)
2.Neutralization assay:Detection of anti-SARS CoV2 neutralizing antibodies by VNT assay: GeneScript kit (n=106)
5.Functional assay: Intracellular cytokine staining for the expression of IFN-γ & CD107a by flowcytometry (n=58)
7.SARS CoV-2 specific 27 plex cytokine assay (n=52)
4.SARS-CoV-2 specific immunophenotyping : Surface staining for B memory, B,T cells and subsets by flowcytometry (n=67)
*5 recovered individuals reported Ist dose of vaccination and hence were removed from further analysis.

## Slide 3
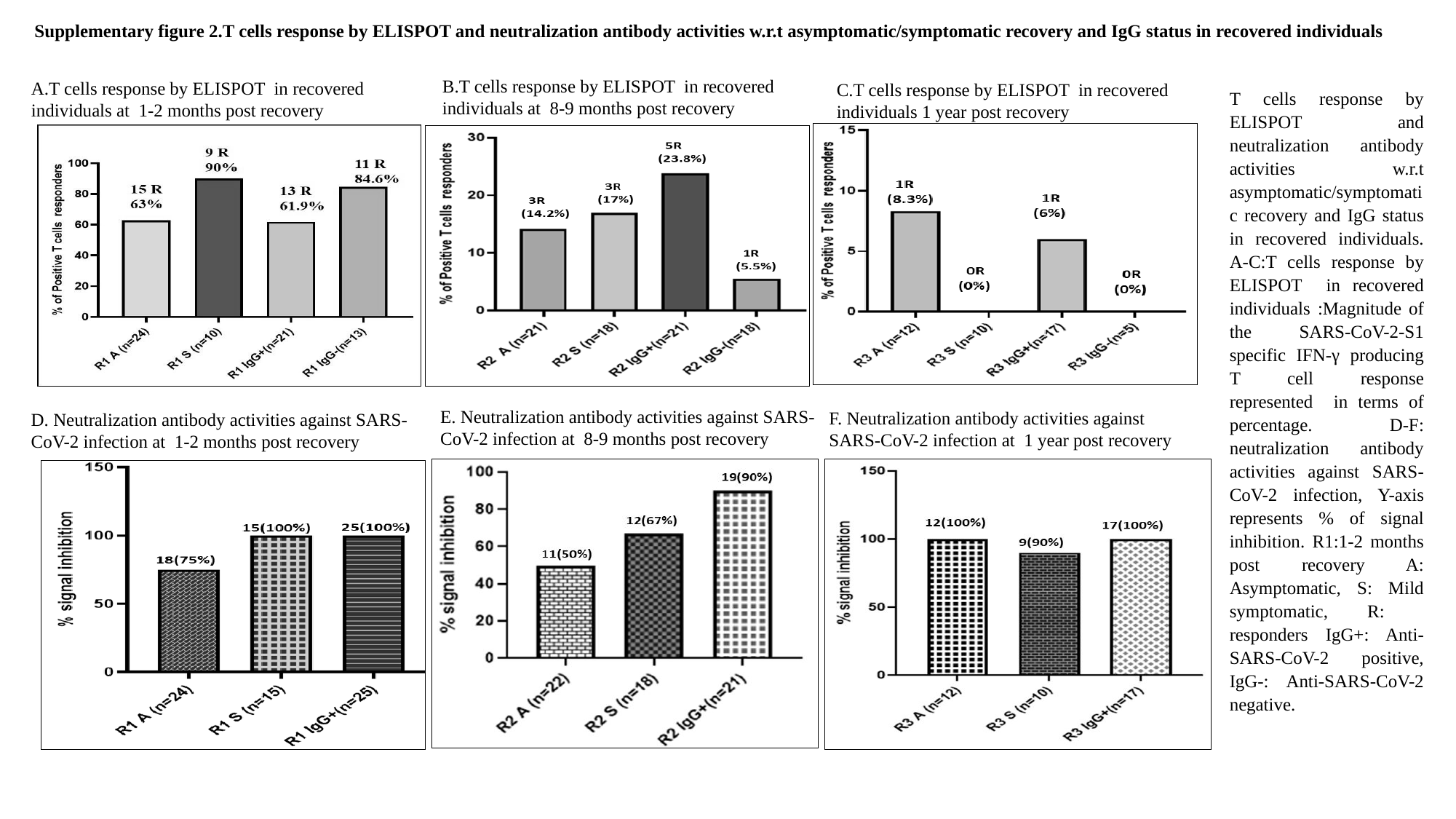

Supplementary figure 2.T cells response by ELISPOT and neutralization antibody activities w.r.t asymptomatic/symptomatic recovery and IgG status in recovered individuals
B.T cells response by ELISPOT in recovered individuals at 8-9 months post recovery
A.T cells response by ELISPOT in recovered individuals at 1-2 months post recovery
C.T cells response by ELISPOT in recovered individuals 1 year post recovery
E. Neutralization antibody activities against SARS-CoV-2 infection at 8-9 months post recovery
F. Neutralization antibody activities against SARS-CoV-2 infection at 1 year post recovery
D. Neutralization antibody activities against SARS-CoV-2 infection at 1-2 months post recovery
T cells response by ELISPOT and neutralization antibody activities w.r.t asymptomatic/symptomatic recovery and IgG status in recovered individuals. A-C:T cells response by ELISPOT in recovered individuals :Magnitude of the SARS-CoV-2-S1 specific IFN-γ producing T cell response represented in terms of percentage. D-F: neutralization antibody activities against SARS-CoV-2 infection, Y-axis represents % of signal inhibition. R1:1-2 months post recovery A: Asymptomatic, S: Mild symptomatic, R: responders IgG+: Anti-SARS-CoV-2 positive, IgG-: Anti-SARS-CoV-2 negative.
